# Cost-effectiveness of edaravone dexborneol versus human urinary kallidinogenase for acute ischemic stroke in China

**DOI:** 10.1186/s13561-024-00479-6

**Published:** 2024-01-29

**Authors:** Pingyu Chen, Mengjie Luo, Yanqiu Chen, Yanlei Zhang, Chao Wang, Hongchao Li

**Affiliations:** 1 School of International Pharmaceutical Business, China Pharmaceutical University, 639 Longmian Road, Jiangning District, Nanjing, 211198 Jiangsu Province China; 2 Center for Pharmacoeconomics and Outcomes Research, China Pharmaceutical University, Nanjing, Jiangsu China; 3 State Key Laboratory of Neurology and Oncology Drug Development (Jiangsu Simcere Pharmaceutical Co.,Ltd., Jiangsu Simcere Diagnostics Co.,Ltd.), Nanjing, Jiangsu China

**Keywords:** Cost Effectiveness, Edaravone dexborneol, Human urinary kallidinogenase, Acute ischemic stroke, Matching adjusted indirect comparison

## Abstract

**Background:**

Clinical trials have demonstrated the efficacy of edaravone dexborneol in the treatment of acute ischemic stroke. This study aims to determine the cost-effectiveness of edaravone dexborneol compared with human urinary kallidinogenase from China’s healthcare system perspective.

**Methods:**

A combination of the decision tree and Markov model was constructed to evaluate the cost-effectiveness of edaravone dexborneol versus human urinary kallidinogenase in the treatment of acute ischemic stroke over a lifetime horizon. Efficacy data were derived from pivotal clinical trials of edaravone dexborneol and human urinary kallidinogenase (TASTE trial and RESK trial, respectively) and adjusted using matching-adjusted indirect comparison. Cost and health utility inputs were extracted from published literature and open databases. One-way deterministic sensitivity and probabilistic sensitivity analyses were performed to examine the robustness of the results.

**Results:**

Compared with human urinary kallidinogenase, edaravone dexborneol generated 0.153 incremental quality-adjusted life years (QALYs) with an incremental cost of ¥856, yielding an incremental cost-effectiveness ratio of ¥5,608 per QALY gained under the willingness-to-pay threshold (one-time gross domestic product per capita). Both one-way deterministic sensitivity analysis and probabilistic sensitivity analysis demonstrated the robustness of the base case results.

**Conclusions:**

Edaravone dexborneol is a cost-effective treatment choice for acute ischemic stroke patients compared with human urinary kallidinogenase in China.

## Introduction

According to the Global Burden of Disease Study 2019, stroke is one of the leading causes of disability and mortality in China and worldwide [1]. Acute ischemic stroke (AIS) is the predominant subtype of stroke, accounting for approximately 80.0% of stroke patients in China [2]. It’s estimated that there were 28.8 million prevalent stroke cases, 3.9 million new cases, and 2.2 million deaths in China in 2019 [3]. Moreover, stroke holds the leading position in the cause of disability-adjusted life-year in China, reaching 45.9 million in 2019^3^. Meanwhile, the total medical cost of AIS has risen to ¥41.7 billion in 2021 and has experienced rapid growth, imposing a heavy burden on families and patients in China [4, 5].

Evidence-based guidelines advocate that early reperfusion therapy including intravenous thrombolysis (IVT) and endovascular therapy (EVT) is the most effective and timely treatment for AIS [2, 6, 7]. However, the high cost and limited accessibility to qualified hospitals hinder the widespread application of IVT and EVT [8, 9]. Consequently, treatment delays in reperfusion therapy significantly compromise the treatment outcomes and hardly meet the clinical needs [8, 10]. In addition to reperfusion therapy, brain cytoprotective and antiplatelet therapy are also recommended as part of comprehensive treatment strategy for AIS by Chinese stroke guidelines [6, 7].

Edaravone dexborneol (EDB), comprised of edaravone and ( +)-bornel, represents a novel cytoprotective medicine. Both pharmacological and clinical studies have shown its antioxidant and anti-inflammatory effects to protect the brain from AIS injury [11–13]. EDB, along with human urinary kallidinogenase (HUK) and butylphthalide, are all under the category of brain cytoprotective medicine and enrolled into the National Reimbursement Drug List (NRDL) to treat AIS in China [14]. However, little is known about the cost-effectiveness of EDB compared with other cytoprotective medicines treating AIS in the NRDL. Among them, HUK has the lowest daily treatment cost. This study explores the cost-effectiveness of EDB compared with HUK in China AIS patients from the Chinese healthcare payers’ perspective, aiming to provide evidence for healthcare decision-making.

## Methods

### Model overview

**Fig. 1:**
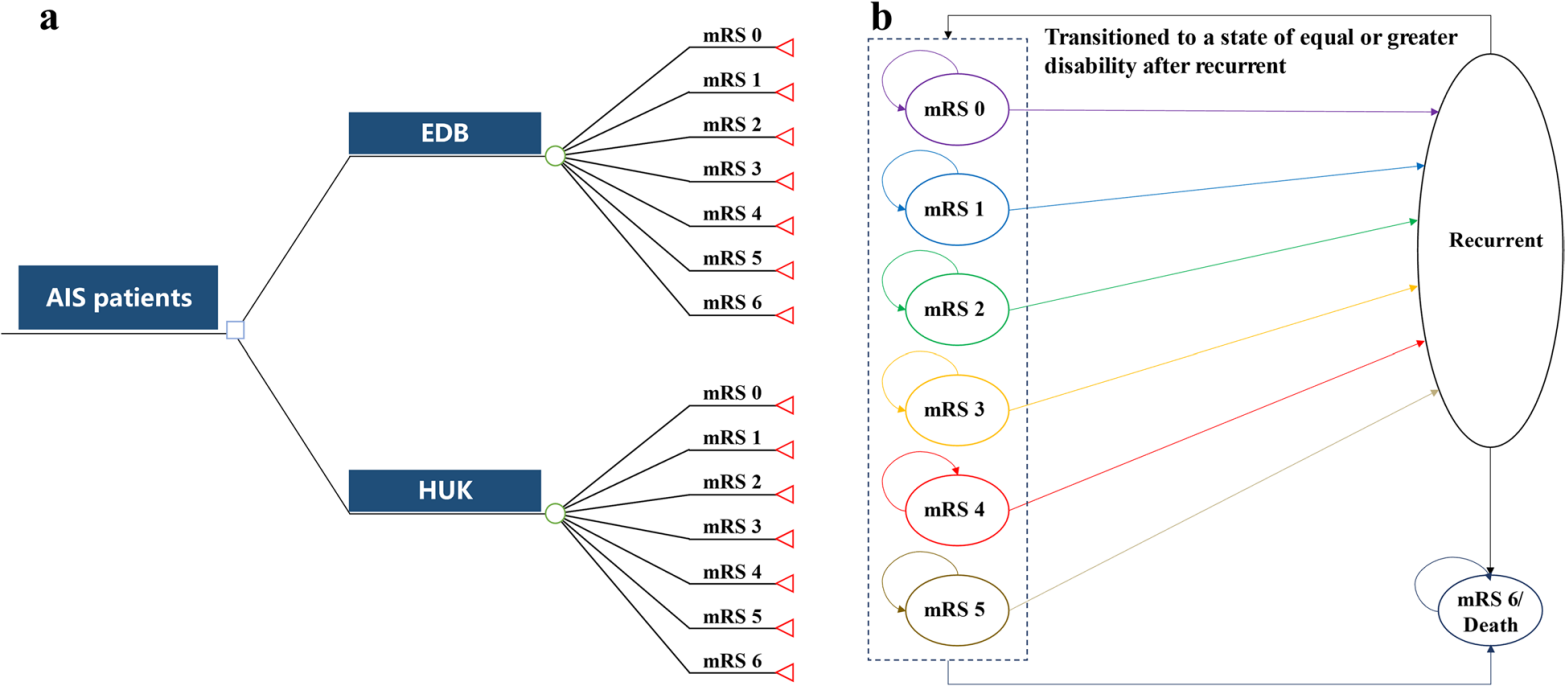
Model structure. **a** Short-term decision tree model; **b** Long-term Markov model. Patients aged 60 with acute ischemic stroke within 48h after stroke onset entered the model and received either edaravone dexborneol or human urinary kallidinogenase. In the decision tree model, patients would distribute among different health states at 90 days and transit to Markov model. Patients may either remain in their current state, experience a recurrent ischemic stroke and transition to a state of equal or greater disability, or die from age-specific mortality or excess mortality

This study was conducted subject to *China guidelines for pharmacoeconomic evaluations (2020 edition)* [15]. This article was prepared according to *Consolidated Health Economic Evaluation Reporting Standards (CHEERS 2022)* reporting guidelines [16]. From China’s healthcare system perspective, a combination of a short-term decision tree and a long-term Markov model (Fig. 1) was constructed using Microsoft Excel 2019 to evaluate the lifetime cost-effectiveness of EDB versus HUK.

A short-term decision tree model (Fig. 1A) was constructed to calculate the costs and health outcomes of patients in the two treatment arms from randomization to the 90th day (D90) after treatment. Seven health states were divided by modified Rankin Scale (mRS) scores 0–6, and the model endpoint was the distribution of mRS scores of patients on D90 after treatment. The mRS score is global outcome rating scale for patients’ post-stroke, and the higher mRS represents more severity and disability. A category of 6 is added as the state of death. According to the clinical trials of EDB and HUK, more than 92.0% of patients were randomized with a baseline mRS score of 0 to 1, so it was assumed that the mRS scores of all the patients initially entered the decision tree model were 0 to 1. However, the specific proportion of patients with mRS 0 and mRS 1 was not reported in HUK clinical trials. Therefore, based on the EDB clinical trial, it was assumed that the proportion of patients initially enrolled in the decision tree with mRS score 0 and mRS score 1 were 90.3% and 9.7%, respectively.

The simulated distribution of health states defined by mRS scores on D90 from the decision tree model was used as the initial health state distribution of the Markov model (Fig. 1B), and the first 90 days were filled up to the first year in the Markov cycle. The cycle length was one year, and the time horizon was 40 years.

At the end of each Markov cycle, the patients either remained in their current state, experienced a recurrent ischemic stroke and further transitioned to a state of equal or greater disability, or died from age-specific mortality or excess mortality. A half-cycle correction was performed with the first half year of cycle 0 included in the calculations. The detailed model structure is shown in Fig. 1.

Costs and health outcomes over a lifetime were calculated and discounted at an annual rate of 5% according to *China guidelines for pharmacoeconomic evaluations* [15].

### Patients and treatments

The target population in the study were adult patients diagnosed with AIS and administered within 48h of stroke onset, with an mRS score of 0 to 2 and a National Institute of Health Stroke Scale (NIHSS) score of between 6 and 25. The characteristics of the target population were assumed to mirror those in TASTE trial and RESK trial [11, 17]. The median age of patients in the two clinical trials was 61 and 62 years, respectively, and an average age of 60 years was assigned to the patients in this study.

The intervention group received EDB intravenous infusion of 37.5 mg/dose (edaravone, 30 mg; ( +)-borneol, 7.5 mg) once every 12 h for 14 continuous days. The control group received HUK (0.15 pNa unit), once a day, which was continued for 21 days.

## Model inputs

### Clinical inputs and transition probabilities

The clinical parameters in the short-term decision tree model were derived from clinical trials of EDB and HUK and were indirectly compared by the unanchored matching-adjusted indirect comparison (MAIC) method. The TASTE trial (NCT02430350) and the RESK trial (NCT02562183), which shared the same primary efficacy outcome of mRS score distribution on D90 after treatment, were included in our study. The phase III trial of HUK was excluded because of small simple size and early publication. The TASTE trial and RESK trial also had similar inclusion and exclusion criteria in age, clinical diagnosis, time limit from stroke onset to drug administration, baseline mRS score and NIHSS score. Detailed information was published previously [11, 17]. Briefly, TASTE is a phase III, randomized, double-blind, comparative trial that enrolled 1,200 patients to compare EDB to edaravone alone in the treatment of AIS, while RESK is a phase IV, single-arm study that included 1,202 AIS patients treated with HUK.

In the absence of a direct head-to-head clinical trial comparing EDB and HUK, an indirect comparison method of health outcome needs to be adopted. Given the availability of the individual patient data (IPD) of TASTE trial and the single-arm nature of the RESK study, unanchored MAIC was applied in this study to compare their shared efficacy indicators [18]. Within unanchored MAIC, treatment effect modifiers and prognostic variables were included as matching variables [18], and thirteen key matching variables of baseline characteristics were chosen from the RESK trial. The differences in these key baseline characteristics between the two trials and the results of matching are shown in Table 1. The IPD of TASTE trial was weighted according to the matching variables by calculating the propensity score. According to the weight allocation of patients in TASTE clinical trials, the proportion of patients with different mRS scores was recalculated to obtain the adjusted distribution proportion of mRS scores [19]. Efficacy outcomes (mRS score distribution on D90 after randomization) before and after MAIC were shown in Table 2. The proportion distribution of mRS scores in EDB group after MAIC was observed to be slightly worse than before MAIC.

**Table 1:** Population difference and MAIC results

	**Before MAIC**		**After MAIC**	
	HUK	EDB	HUK	EDB
Age, year; Median	62.00	62.96	62.00	61.47
Men, %	68.05	67.45	68.05	68.05
Body mass index, kg/m^2^; Median	24.01	24.20	24.01	24.05
Smoke, %	43.30	38.90	43.30	43.30
Time to treatment, h; Median	30.5	28.0^a^	30.50	30.47
Previous stroke, %	27.12	29.05	27.12	27.12
Baseline mRS(0–1), %	92.06	99.83	92.06	92.06
NIHSS score^b^, Median	7.00	6.00	7.00	6.63
Cardioembolic, %	0.75	5.18	0.75	0.75
Hypertension, %	66.31	65.11	66.31	66.31
Diabetes, %	30.53	25.21	30.53	30.53
Hyperlipidemia, %	14.89	7.35	14.89	14.89
Coronary heart disease, %	11.20	8.68^a^	11.20	11.20

*EDB* Edaravone dexborneol, *HUK* Human urinary kallidinogenase, *mRS* modified Rankin Scale, *MAIC* Matching-adjusted indirect comparison, *NIHSS* National Institutes of Health Stroke Scale

^a^Time to treatment and proportion of coronary heart disease of EDB group were extracted from the clinical study report

^b^The higher NIHSS score indicates more disease severity

**Table 2:** Distribution of mRS score on D90 after randomization before and after MAIC

Before MAIC			After MAIC		
	HUK	EDB		HUK	EDB
mRS 0	19.0%	22.7%	mRS 0	19.0%	20.2%
mRS 1	37.5%	44.4%	mRS 1	37.5%	40.6%
mRS 2	17.8%	12.8%	mRS 2	17.8%	12.0%
mRS 3	16.0%	10.9%	mRS 3	16.0%	19.3%
mRS 4	8.3%	6.3%	mRS 4	8.3%	4.8%
mRS 5	1.3%	2.7%	mRS 5	1.3%	3.1%
mRS 6	0.1%	0.0%	mRS 6	0.1%	0.0%

*D90* 90 days, *EDB* Edaravone dexborneol, *HUK* Human urinary kallidinogenase, *mRS* modified Rankin Scale, *MAIC* Matching-adjusted indirect comparison

The transition probabilities in the long-term Markov model were derived from the literature. The excess death in AIS patients relative to the general population was considered, and the mortality rates of ischemic stroke patients were adjusted with mRS state-specific hazard ratio (HR) from a published study [20]. The age-specific mortality rate for the general population was drawn from the China life Table [21]. The recurrent rate of stroke is age-dependent according to previous literature [8–10, 22], and the risk of recurrent stroke in this study was assumed to be the same across all mRS health states for both groups and was extracted from a stroke registry study [23]. The distribution of patients after a recurrent stroke was obtained from the study of Gao Lan [24] (Tables 3 and 4).

**Table 3:** Distribution after recurrent stroke

		**mRS state before recurrent**						**Reference**
		mRS 0	mRS 1	mRS 2	mRS 3	mRS 4	mRS 5	
**Post-recurrent mRS state**	mRS 0	17.82%						[24]
	mRS 1	22.77%	40.59%					
	mRS 2	8.91%	8.91%	49.50%				
	mRS 3	11.88%	11.88%	11.88%	61.38%			
	mRS 4	13.86%	13.86%	13.86%	13.86%	75.24%		
	mRS 5	6.93%	6.93%	6.93%	6.93%	6.93%	82.17%	

*mRS* modified Rankin Scale

**Table 4:** Model input parameters

Parameters	Base case value	Lower value	Upper value	Distribution type	Reference
**Cost (2021, Chinese Yuan Renminbi)**					
Hospital days	9.9	/			[4]
Edaravone dexborneol	33	33	49	/	NRDL
Human urinary kallidinogenase	100	90	100	/	[25]
Hospitalization costs for mRS (0–2)	12,614	12,428	12,802	gamma(α = 17494.59;β = 0.72)	[31]
Hospitalization costs for mRS (3–5)	17,223	16,845	17,606	gamma(α = 7860.91;β = 2.19)	
Hospitalization costs for mRS (6)	13,951	12,819	15,155	gamma(α = 548.15;β = 25.45)	
Post-stroke costs for mRS (0–2)	9,264	8,977	9,558	gamma(α = 3909.41;β = 2.37)	
Post-stroke costs for mRS (3–5)	14,238	13,460	15,049	gamma(α = 1235.08;β = 11.53)	
**Health utility**					
mRS 0	0.95	0.90	1.00	beta(α = 56315.05;β = 2963.95)	[27]
mRS 1	0.89	0.78	1.00	beta(α = 10469.23;β = 1293.95)	
mRS 2	0.67	0.48	0.86	beta(α = 803.62;β = 395.81)	
mRS 3	0.44	0.20	0.68	beta(α = 172.72;β = 219.83)	
mRS 4	0.16	0.00	0.35	beta(α = 126.72;β = 665.28)	
mRS 5	0.10	0.00	0.28	beta(α = 7.68;β = 69.10)	
Disutility of stroke	0.086	0.060	0.112	beta(α = 38.33;β = 407.36)	
**Hazard ratios for mortality relative to the general population**					
mRS 0	1.53	1.23	1.83	lognormal(α = 0.43;β = 0.10)	[20]
mRS 1	1.52	1.20	1.83	lognormal(α = 0.42;β = 0.11)	
mRS 2	2.17	2.14	2.20	lognormal(α = 0.77;β = 0.01)	
mRS 3	3.18	3.17	3.19	lognormal(α = 1.16;β = 0.002)	
mRS 4	4.55	4.31	4.78	lognormal(α = 1.52;β = 0.03)	
mRS 5	6.55	6.12	6.98	lognormal(α = 1.88;β = 0.03)	
**China Life Table (natural mortality rate, per year)**					
60–64 years	0.75%	/			[21]
65–69 years	1.17%				
70–74 years	2.03%				
75–79 years	3.56%				
80–84 years	6.29%				
85–89 years	10.28%				
90 years or older	16.17%				
**Stroke recurrent rate**					
Recurrent rate (0–1 year)	5.9%	/			[23]
Recurrent rate (1–2 year)	3.6%				
Recurrent rate (2–3 year)	2.5%				
Recurrent rate (3–4 year)	2.2%				
Recurrent rate (4–5 year)	2.2%				
Recurrent rate (5–6 year)	2.7%				
Recurrent rate (6–7 year)	2.7%				
Recurrent rate (7–8 year)	2.3%				
Recurrent rate (8–9 year)	2.8%				
Recurrent rate (9 year and above)	1.6%				
**Others**					
Discount rate of cost	0.05	0.00	0.08	/	[15]
Discount rate of health outcome	0.05	0.00	0.08		

*mRS* modified Rankin Scale, *NRDL* National reimbursement drug list

### Costs

From China’s healthcare system perspective, only direct medical costs were considered, including drug costs, hospitalization costs, and post-stroke costs for secondary prevention. The prices of EDB and HUK were obtained from NRDL and MENET database (https://www.menet.com.cn/) [25], respectively. The hospitalization days were assumed to be the same across the treatment arms and obtained from the *China Healthcare Statistical Yearbook 2021* [4]. One-time hospitalization costs and post-stroke costs stratified by mRS score were extracted from the China National Stroke Registry [26]. All costs were converted to 2021 Chinese Yuan (¥) using the medical care component of China’s Consumer Price Index (Table 4).

### Health utilities

The health outcome in this study was represented by quality-adjusted life years (QALYs). The utility scores of each mRS health state were derived from the CHANCE trial (NCT00979589) [27], which applied EuroQol-Five Dimension (EQ-5D) and Chinese preference weights to calculate health utilities. In addition, our study considered the disutility of stroke attack and assumed the disutility of AIS was consistent with the annual disutility of cerebrovascular disease for type 2 diabetes patient in the study by Mok CH [28] (Table 4).

### Cost-effectiveness analysis

Incremental cost-effectiveness ratio (ICER) and net monetary benefit (NMB) were used to compare the cost-effectiveness of EDB. As recommended by the *China guidelines for pharmacoeconomic evaluations* [15], one-time gross domestic product (GDP) per capita was chosen as the willingness to pay (WTP) threshold, i.e. ¥80,976 per QALY in China in 2021. NMB, defined as QALY*WTP-cost, was also reported and used to compare the two arms in the sensitivity analysis.

### Sensitivity analysis

Both deterministic sensitivity analysis (DSA) and probabilistic sensitivity analysis (PSA) were performed to explore the robustness of the results. A one-way DSA was undertaken by varying one parameter between a given range while keeping others fixed (Table 3). PSA was implemented by assigning a certain distribution to key parameters, and second-order Monte Carlo simulation (1,000 iterations) was performed with parameters sampled from the defined distributions (Table 3). In accordance with previous AIS cost-effectiveness studies [8, 9, 24], costs, health utilities, and HRs were assumed to follow a gamma distribution, beta distribution, and lognormal distribution, respectively. A tornado diagram was used to present the results of one-way DSA, while a scatterplot and a cost-effectiveness acceptability curve were used to present the results of PSA.

## Results

### Base-case analysis

The costs and health outcomes in the base-case analysis were summarized in Table 5. After 40 cycles of the Markov model, more than 99.7% of patients in both groups died. In the lifetime (40 years) horizon, the EDB group generated 0.153 incremental QALYs and ¥856 incremental cost, yielding an ICER of ¥5,608 per QALY gained. Under the threshold of ¥80,976 per QALY, the NMB of EDB was higher than that of HUK in the base-case analysis (¥588,426 versus ¥576,927, respectively). The incremental NMB was ¥11,499, indicating that EDB was likely to be a cost-effective option in treating AIS.

**Table 5:** Base-case costs and health outcomes results

	**EDB**	**HUK**
QALYs	7.621	7.468
Costs	¥28,687	¥27,832
Drug costs	¥2,772	¥2,100
Hospitalization costs	¥18,116	¥18,005
Post-stroke costs	¥7,799	¥7,727
NMB	¥588,426	¥576,927
Incremental costs	¥856	
Incremental QALYs	0.153	
Incremental NMB	¥11,499	
ICER	¥5,608	

*ICER* Incremental cost-effectiveness ratio, *NMB* Net monetary benefit, *QALY* Quality-adjusted life year

### Sensitivity analysis

**Fig. 2:**
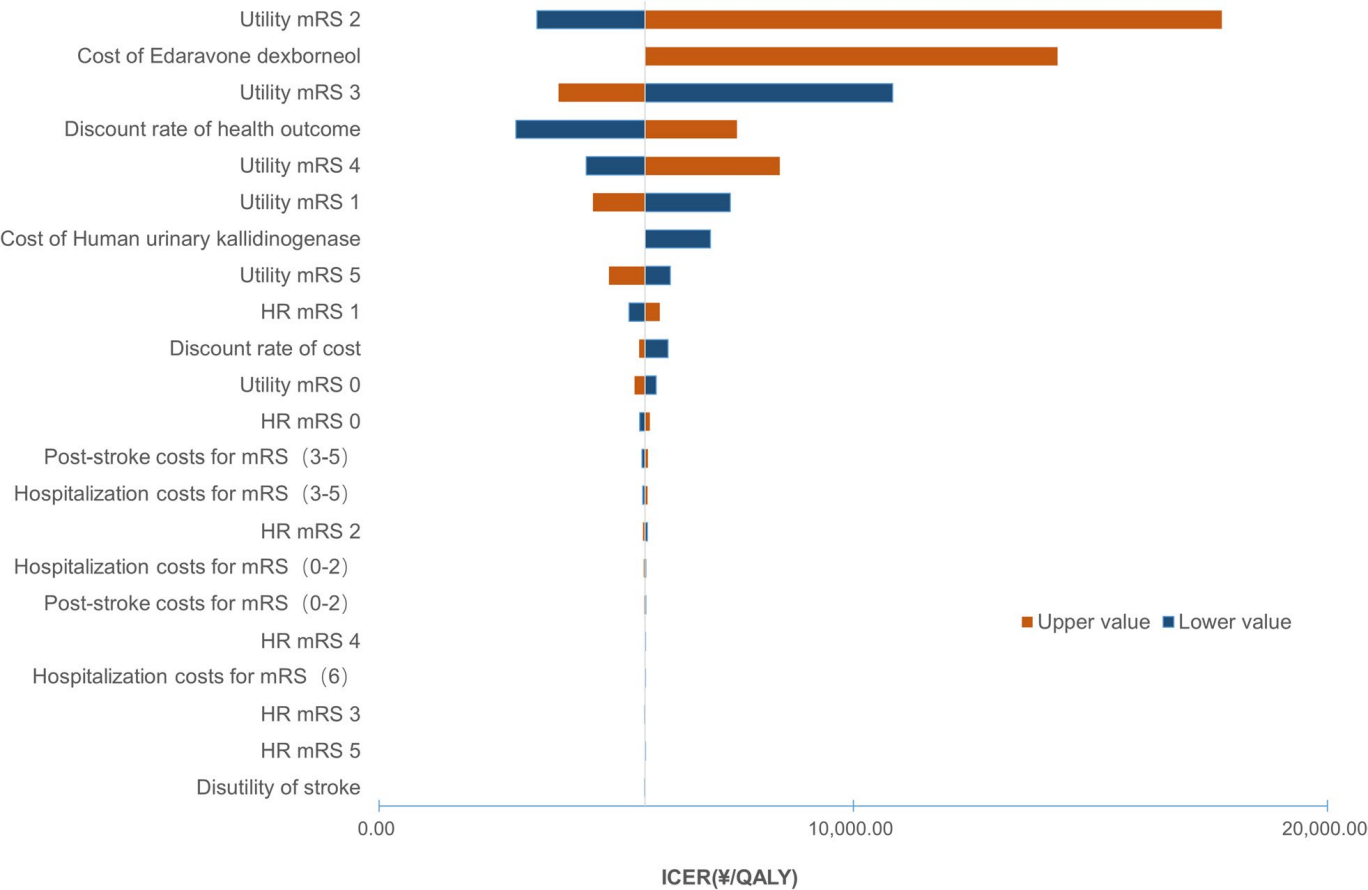
Tornado diagram. *HR* hazard ratio, *ICER* incremental cost-effectiveness ratio, *mRS* modified Rankin Scale, *QALY* quality-adjusted life year

As shown in Fig. 2, the results were most sensitive to the health utility of mRS2. When the utility of mRS2 ranged between 0.48 to 0.86, the corresponding ICER varied between ¥3,329 and ¥17,769 per QALY. The ICERs of one-way sensitivity analyses of all parameters were below the threshold of ¥80,976 per QALY, demonstrating the robustness of the base-case results.

**Fig. 3:**
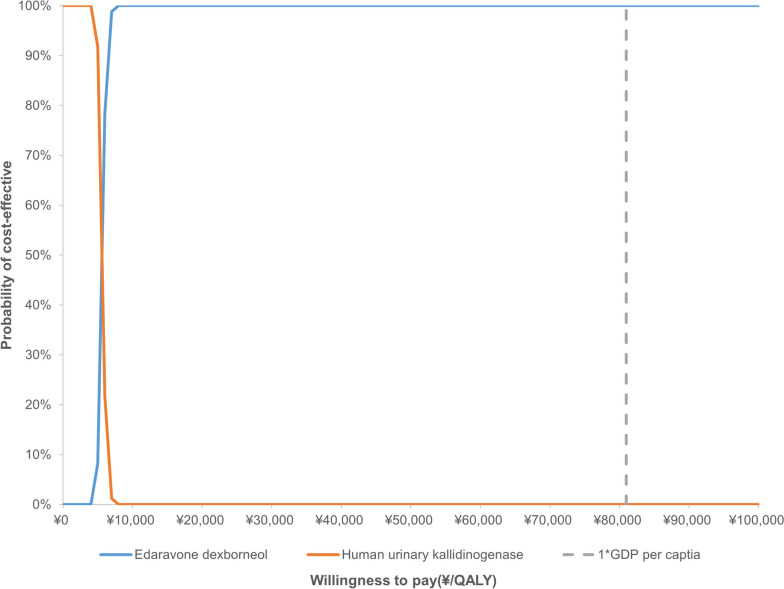
Cost-effectiveness acceptability curves. *GDP* gross domestic product, *QALY* quality-adjusted life year

**Fig. 4:**
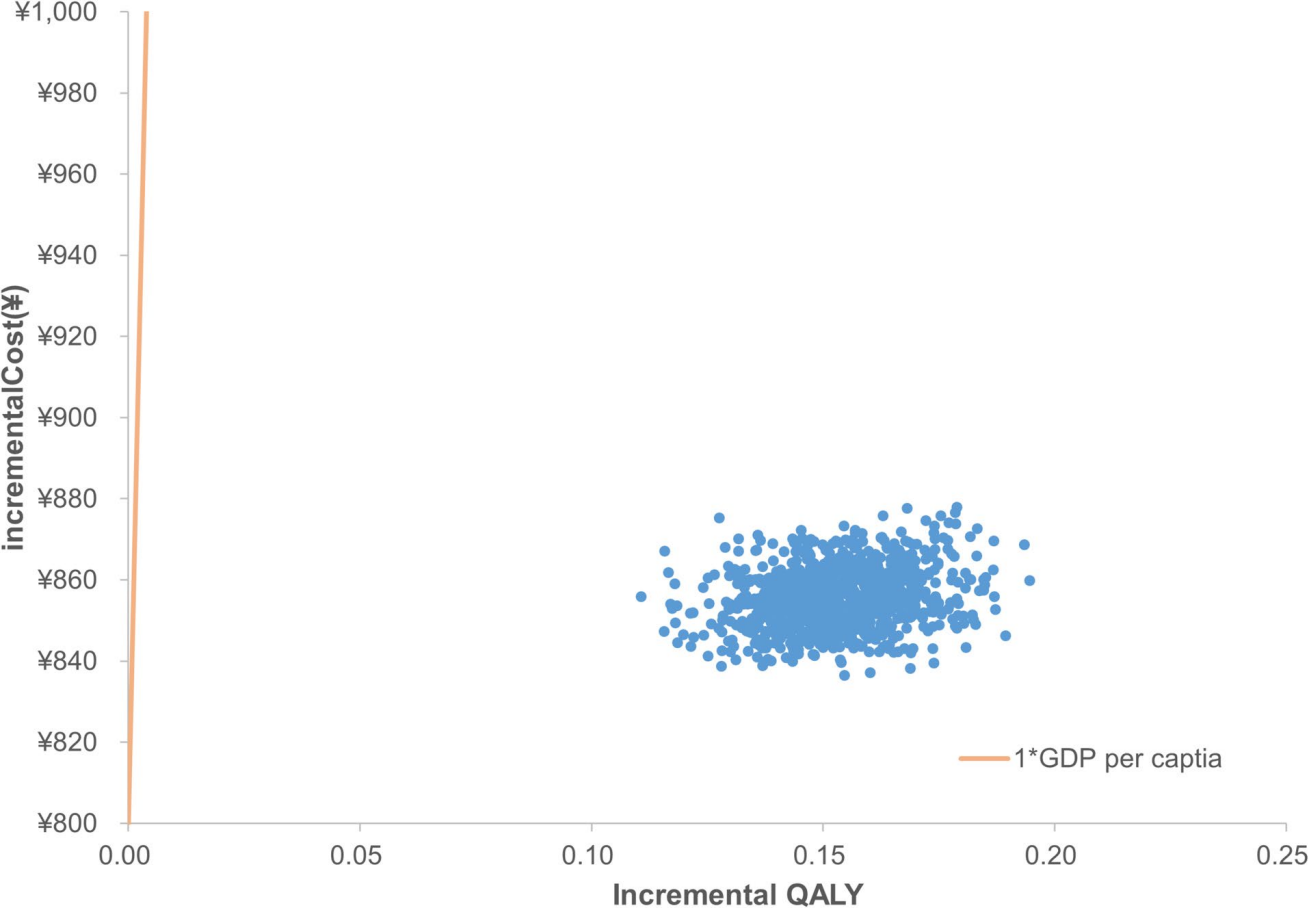
Incremental cost-effectiveness ratio scatterplot. *GDP* gross domestic product, *QALY* quality-adjusted life year

The PSA results with a lifetime (40 years) horizon were reported in Figs. 3 and 4. According to the cost-effectiveness acceptability curve, the probability of EDB being cost-effective was 100% under the WTP threshold of ¥80,976 per QALY.

## Discussion

This study compared the cost-effectiveness of EDB versus HUK based on the MAIC results of pivotal clinical trials. The findings suggested that EDB demonstrated the lifetime cost-effectiveness compared with HUK in the treatment of AIS patients from China’s healthcare system perspective.

Although early reperfusion therapy including IVT and EVT is recognized as the gold standard for AIS treatment, the timing of treatment initiation significantly influences the clinical outcomes and cost-effectiveness [8]. However, due to limitations in medical resources and socioeconomic status factors, the overall rate of IVT and EVT between 2019 and 2020 were 5.6% and 1.5%, respectively [29]. Beyond reperfusion therapy, cytoprotective agents are another effective treatment for AIS. EDB, combined edaravone and ( +)-borneol, has shown apparent efficacy in the clinical trial [11].

Leveraging the unanchored MAIC results between IPD from the TASTE and RESK trial, the efficacy outcome of mRS score distribution at D90 was compared between EDB and HUK (Table 2). This study combined the decision tree and Markov model to evaluate the long-time cost-effectiveness of EDB in the treatment of China AIS patients. To the best of our knowledge, this is the first study to evaluate the cost-effectiveness of EDB versus HUK in the treatment of AIS from China’s healthcare system perspective. The study incorporates the most recent clinical trial data and employs the MAIC method to enhance its methodological robustness. While recognizing the importance of direct comparisons, these findings can offer valuable insights into clinical decision-making until direct comparative evidence becomes available.

Limitations of this evaluation should be considered when interpreting these results. First of all, both anchored or unanchored MAIC methods have a key assumption that treatment effect modifiers and prognostic variables are consistent across the treatment populations [18]. However, the challenge lies in the potential inability to include all relevent effect modifiers and prognostic variables. Besides, unanchored MAIC is considered less robust than the anchored method due to the stronger assumptions [30]. Notably, apart from the single-arm RESK trial, there are no high-quality trials with a large sample available for HUK, aside from the single-arm RESK trial, limits the strength of the comparison. However, the two trials share the similarity in study design, patient inclusion and exclusion criteria, and results report form, the unanchored MAIC is the best available method that could be applied to compare EDB and HUK. Head-to-head studies evaluating EDB versus HUK are recommended to validate and confirm these results in the future.

This study shared a similar model structure and cost sources with another RCT-based economic evaluation that demonstrated the cost-effectiveness of EDB versus edaravone alone in the treatment of Chinese AIS patients [10], and variations in the calculation of recurrence rate and death rate exist. Since the daily treatment cost of edaravone is much lower than HUK, our study suggests a high likelihood of cost-effectiveness for EDB for AIS patients in China.

Secondly, due to lack of high-quality studies specific in Chinese population, the extraction of transition probabilities and clinical data from other countries may introduce potential heterogeneity. The unclear association between risk of recurrent stroke and mRS score led to the assumption in this study that the risk of recurrent stroke is independent of the mRS score, which needs to be validated through further researches. The hospitalization costs and post-stroke costs were derived from the registry study, but the timeliness of date may result in uncertainty of cost parameters. The disutility of stroke attack was obtained from the disutility of cerebrovascular disease for type 2 diabetes patients. Therefore, sensitivity analysis was conducted in this study, and the results were robust.

Furthermore, this study was performed from China’s healthcare system perspective and did not calculate the indirect cost. However, the loss of caregiver and patient productivity may lead to additional indirect costs other than direct medical costs. Adverse event (AE) costs were omitted since the low incidence of severe AEs in both trials, and the exclusion of AE costs may not significantly impact the results.

Last but not least, some patients in the RESK trial may have received combination treatments with other drugs for less than 21 days. In contrast, patients in the TASTE trial were all treated with EDB alone for 14 days. This difference may potentially lead to an underestimation or overestimation of the results.

This study demonstrated the long-term cost-effectiveness of EDB compared with HUK in the treatment of acute ischemic stroke patients, from China’s healthcare system perspective. These findings could offer valuable insights for guiding practical decisions on the allocation of medical resources.

## Data Availability

All data analyzed during this study were available in this article, further inquiries can be directed to the corresponding author.

## Abbreviations

AIS: Acute ischemic stroke
D90: The 90th day
DSA: Deterministic sensitivity analyses
EDB: Edaravone dexborneol
EVT: Early endovascular therapy
GDP: Gross domestic product
HR: Hazard ratio
HUK: Human urinary kallidinogenase
ICER: Incremental cost-effectiveness ratio
IPD: Individual patient data
IVT: Intravenous thrombolysis
MAIC: Matching-adjusted indirect comparison
mRS: Modified Rankin Scale
NIHSS: National Institute of Health Stroke Scale
NMB: Net monetary benefit
NRDL: National reimbursement drug list
PSA: Probabilistic sensitivity analyses
WTP: Willingness to pay
